# Extreme wealth loss and subsequent mental well-being among adults aged ≥50 years: a multicohort study

**DOI:** 10.7189/jogh.16.04156

**Published:** 2026-09-11

**Authors:** Ke Peng, Minzhi Xu, Yun Liu, Yanhong Gong

**Affiliations:** 1 Department of Social Medicine and Health Management, School of Public Health, Tongji Medical College, Huazhong University of Science and Technology, Wuhan, Hubei, China; 2 Otolaryngology-Head and Neck Surgery, Department of ENT, Tongji Hospital, Tongji Medical College, Huazhong University of Science and Technology, Wuhan, Hubei, China

**Keywords:** extreme wealth loss, mental well-being, longitudinal study, cross-national study

## Abstract

**Background:**

The association of extreme wealth loss with subsequent mental well-being in later life, as well as whether this association varies across countries with different levels of economic development, remains unclear.

**Methods:**

Our study included 23,817 participants, of which 3282 were from the China Health and Retirement Longitudinal Study (2011–2018), 11,838 from the Health and Retirement Study (2008–2019), and 8697 from the Mexican Health and Aging Study (2012–2019). Extreme wealth loss in our study is defined as a net wealth decline of 75% or more between the first two waves. Mixed-effects linear regression models were employed to examine the associations.

**Results:**

In the fully adjusted models, extreme wealth loss was associated with higher depressive symptoms (odds ratio (OR) = 1.3; 95% confidence interval (CI) = 1.2–1.4 for China, OR = 1.3; 95% CI = 1.2–1.3 for USA, and OR = 1.1; 95% CI = 1.0–1.1) for Mexico); lower life satisfaction (OR = 0.8; 95% CI = 0.7–0.9) for China, OR = 0.8; 95% CI = 0.7–0.8) for USA, OR = 0.9; 95% CI = 0.9–1.0 for Mexico) and worse self-reported health (OR = 0.9; 95% CI = 0.8–0.9 for China, OR = 0.9; 95% CI = 0.8–0.9 for USA, OR = 1.0; 95% CI = 0.9–1.1 for Mexico). Predicted standardised scores showed that the negative association between extreme wealth loss and mental well-being remained unchanged over time in China and Mexico. In the USA, we observed a significant interaction between exposure groups and follow-up time.

**Conclusions:**

Public health policy formulation should target populations experiencing significant wealth loss and provide them with stable economic security to effectively enhance mental health outcomes.

Well-being is the foundation of a healthy life. However, the prevalence of mental disorders is rising rapidly worldwide across all age groups, particularly among middle-aged and older individuals [1]. Approximately 14% of the population suffers from mental disorders, such as depression, which accounts for an estimated 2.7% of disability-adjusted life years for this demographic globally [2]. Most of the causes of mental disorders have social origins, including socioeconomic status (SES), social resources, and social relationships, among which SES is conceptualised as a fundamental cause [3,4]. Disadvantaged socioeconomic groups are often in poor social resources and social relationship environments, unable to obtain adequate health protection, leading to disease and death [5,6].

Wealth is the most direct indicator of SES, and evaluating its impact – especially the effects of wealth loss – on health is particularly important [7]. Not only is wealth associated with improved access to health services, but certain components of wealth, such as income, can be directly influenced by policy interventions [8]. Epidemiological studies have demonstrated that consistently low hourly wages are significantly linked to higher all-cause mortality rates [9]. Additionally, a sudden decrease in wealth may elevate the risk of cardiovascular disease, highlighting the substantial influence of wealth on health outcomes [10]. However, important gaps remain regarding the association between wealth shocks and mental well-being. First, a meta-analysis indicates that current research on the relationship between changes in wealth, as measured by income, and mental health and well-being is uncertain due to a high risk of bias and unmeasured confounding variables [11]. Second, existing studies primarily focus on the short-term effects of wealth shocks on mental health, typically spanning less than two years. For example, Gardner and Oswald found that a financial windfall, measured by medium-sized lottery winnings, was significantly associated with improved mental well-being two years later [12]. Separately, Osypuk and colleagues showed that women who had recently experienced foreclosure within the previous two years had a higher risk of severe depressive symptoms [13]. The long-term potential role of wealth shocks remains largely unknown. Cumulative advantage (disadvantage) theory and life course theory emphasise that socioeconomic inequalities and early adverse circumstances may accumulate over time, forming long-term health trajectories that lead to heterogeneity in individual health and contribute to health inequalities [14]. Understanding the long-term effects of wealth shocks on mental well-being may further guide and improve wealth-related health strategies and practices to promote public mental health. Third, the studies concentrated on individual countries [12,15,16]. Given the variations in definitions and methodologies across these studies, along with the differing contexts of social safety nets in developed and developing countries, it remains uncertain whether older individuals from diverse cultural backgrounds experience wealth shocks in a uniform manner.

As global population ageing and macroeconomic uncertainty rise in parallel, economic insecurity has become a shared concern for academia and policymakers regarding population health, particularly the psychological well-being of older age groups. Existing discussions on ‘wealth and health’ predominantly focus on cross-sectional or long-term associations between wealth levels and health, while research on wealth shocks as dynamic impacts and their heterogeneity across different socio-economic systems and cultural contexts remains scarce. This study employs a unified measurement framework to compare longitudinal cohorts across multiple-nations, this study was therefore designed to harmonised measures of wealth and mental well-being among adults aged 50 years and older across three longitudinal studies. It aims to explore the relationship between extreme wealth loss and subsequent mental health outcomes, the direction of this association, and further investigates the persistence or recovery of this impact under differing institutional contexts.

**Adherence to JoGH’s Guidelines for Reporting Analyses of Big Data Repositories Open to the Public (GRABDROP)**

This study was based on secondary analyses of harmonised longitudinal ageing cohort data. We followed JoGH’s Guidelines for Reporting Analyses of Big Data Repositories Open to the Public (GRABDROP) [17]. Details are provided in the **Online Supplementary Document**. Briefly, this study was hypothesis-driven and examined the association between extreme wealth loss and subsequent mental well-being among adults aged 50 years and older. We reviewed relevant previous studies using the same or related data sets and cited them where appropriate. We also clarified the distinction between the primary analysis, sensitivity analyses, and subgroup analyses, and interpreted secondary and exploratory findings cautiously. No AI tools were used for data analysis. Any use of language-editing assistance did not affect the study design, statistical analysis, interpretation of results, or scientific conclusions, all of which were verified by the authors.

## METHODS

### Study design and participants

For this population-based longitudinal study, data were analysed from US Health and Retirement Study (HRS) (2008–2019, survey of adults aged ≥51 years) and its harmonised international partner studies: the China Health and Retirement Longitudinal Study (CHARLS; (2011–2018, survey of adults aged ≥45 years) and the Mexican Health and Aging Study (MHAS) (2012–2019, survey of adults aged ≥50 years). The data collection dates are reported by year, and additional details can be found in the published cohort profiles [18–20]. The study population consisted of a total of 23,817 individuals: 3282 from China, 11,838 from the USA, and 8697 from Mexico. We limited our study to adults aged 50 years and older at baseline to ensure age consistency across countries. Participants who lacked information about wealth during the first two waves, did not reassess the outcomes in any subsequent surveys, or had missing baseline covariates were excluded from the analysis (Figure S1 in the **Online Supplementary Document****)**. An overview of the data sets, time points, exposures, outcomes, and covariates for each cohort was provided (Table S1 in the **Online Supplementary Document****)**.

All cohorts received ethical approval from the relevant local research ethics committees. All participants were recruited after providing written informed consent. HRS was approved by the University of Michigan Institutional Review Board. CHARLS obtained approval from the Biomedical Ethics Review Committee of Peking University. MHAS was approved by the Ethics Committee of the University of Texas Medical Centre.

### Exposure assessment

The exposure was extreme household wealth loss during specific periods: from 2011 to 2013 in China (waves 1–2), from 2008 to 2011 in the USA (waves 9–10), and from 2012 to 2015 in Mexico (waves 3–4), which aimed to capture substantial wealth changes occurring between adjacent survey waves. In each country, wealth was assessed using couple-level wealth variables, which represent the total net worth of assets owned by an individual and their spouse or partner. These variables were derived from the RAND HRS Longitudinal File and the harmonised CHARLS and MHAS data sets, which are accessible on the Gateway to Global Aging Data platform (https://g2aging.org/survey/overview). The components used to calculate wealth for each country (Table S2 in the **Online Supplementary Document****)**. This approach is consistent with previous eight longitudinal studies examining the health consequences of major wealth shocks. Treating extreme wealth loss as a time-invariant exposure factor to examine its subsequent association with mental health trajectories during follow-up.

Wealth is expressed in real 2010 values by adjusting for inflation using each country’s consumer price index (CPI). Extreme wealth loss in our study is defined as a net wealth decline of 75% or more between the first two waves. This definition has been demonstrated in previous studies to correlate with the health status of individuals during significant economic shocks [15,21,22]. In addition, to capture the association between the gradient of wealth loss and mental well-being in a finer granularity, we further categorised wealth loss into five groups:

• no wealth loss;

• wealth loss (0 − 25%);

• wealth loss (25 − 50%);

• wealth loss (50 − 75%);

• wealth loss (75% and above).

### Covariates

We considered possible confounding factors, including sociodemographic factors (age (continuous), sex (male/female), marital status (married/unmarried), household size (continuous), and contact with others (weekly contact/less than weekly); socioeconomic status (education (primary/sary/tertiary), and labour force status (working/retired/others)); health behaviours (current smoking (yes/no) and alcohol consumption (less than weekly alcohol use/weekly)); and physical health (number of chronic conditions (continuous), activities of daily living (ADL) disability (yes/no), and number of limitations on instrumental activities of daily living (IADL) (continuous)), all measured at baseline. The harmonisation of covariates across three longitudinal studies has illustrated (Table S3 in the **Online Supplementary Document**).

### Outcome

The analysis focused on three types of mental well-being: depressive symptoms, life satisfaction, and self-reported health. The measurement items and their response options varied slightly across countries (Table S3 in the **Online Supplementary Document**). These three outcomes were selected because recent research broadly agrees that mental health should be viewed as a continuum, ranging from well-being (*e.g.* self-reported health and life satisfaction) to mental illness (*e.g.* depressive symptoms), rather than focusing solely on mental disorders [23–25]. This concept emphasises the necessity of not only preventing mental disorders but also enhancing overall mental well-being. Mental well-being outcomes were treated as time-varying variables. To ensure comparability across countries, all outcome measures were harmonised and recorded using a standardised framework [23,24]. Depressive symptoms were assessed using Center for Epidemiologic Studies Depression Scale (CES-D), with responses summed to generate a continuous score; higher scores indicated more severe depressive symptoms. Life satisfaction was measured using a single self-report item rating overall satisfaction with life on a numerical scale, with higher scores reflecting greater life satisfaction. Self-reported health was assessed using a standard question asking respondents to rate their overall health status (excellent, very good, good, fair, or poor), with higher values indicating better perceived health.

### Statistical analysis

Baseline characteristics were described using means ± standard deviation (SD) for continuous variables and proportions for categorical variables, categorised by each country.

Given the longitudinal nature of the data with repeated outcome measures, we employed a multivariable-adjusted mixed-effects linear regression model with person-specific random intercepts to examine the association between extreme wealth loss and subsequent mental well-being in each country. Years since the baseline was used as the timescale. We included an interaction term between extreme wealth loss and years since baseline to examine the subsequent changes in three outcomes over time. All models were subsequently adjusted for age, sex, marital status, household size, contact with others, education, labour force status, current smoking, alcohol consumption, chronic conditions, ADL and IADL. The equation for the linear mixed model can be found in the Supplementary Methods in the **Online Supplementary Document**. Taking into account potential differences among subgroups, we conducted separate analyses for sub-populations stratified by age, sex, marital status, social contact, education, and labour force status. A causal diagram illustrating our hypothesised relationship and the corresponding assumptions (Figure S2 in the **Online Supplementary Document**).

Several sensitivity analyses were performed. First, we used inverse probability of treatment weights (IPTW) to address potential confounding arising time-varying covariates. IPTW constructs a weighted pseudo-population in which measured covariates are balanced across exposure groups, thereby reducing confounding and allowing estimation of marginal effects under the assumption of no unmeasured confounding [26]. Second, we excluded participants with memory-related and psychological disorders at baseline and repeated the analysis. Third, a continuous variable was created to represent changes in wealth quintile rankings over the first two waves, with a range from −4 to 4. This variable reflects more nuanced changes in an individual’s relative wealth position within their country over time [21]. Fourth, we repeated the analyses using inverse probability of censoring weights and multiple imputation with chained equations to minimise potential selection bias resulting from selective attrition during follow-up and missing covariates (Supplementary Methods in the **Online Supplementary Document**).

*R*, version 4.0.5 (R Foundation for Statistical Computing) was used for all statistical analyses. Two-sided *P* < 0.05 was considered statistically significant for all analyses.

## RESULTS

### Baseline characteristics

A total of 23,817 participants were included in this analysis (**Table 1**). The average age differed among the countries, with China reporting the youngest mean age at 60.56 years. In contrast, the USA and Mexico had older populations, with mean ages of 67.68 years and 63.48 years, respectively. Among the three cohorts, China had the highest proportion of males (50.15%). The vast majority of participants in China (90.59%) and Mexico (86.93%) had only a primary education, whereas participants in the USA had higher levels of education. Additionally, Mexico had the largest average household size (4.46), while the USA had the smallest (2.16). Extreme wealth loss was less common among older adults in the USA (9.99%) than among their counterparts in China (16.15%) and Mexico (16.98%).

**Table 1:** Participant characteristics by country

Characteristics	China (n = 3282)	USA (n = 11,838)	Mexico (n = 8697)
Age, mean (SD)	60.56 (6.94)	67.68 (9.03)	63.48 (8.34)
Male, n (%)	1646 (50.15)	4718 (39.85)	3539 (40.69)
Unmarried, n (%)	354 (10.79)	3897 (32.92)	2446 (28.12)
Household size, mean (SD)	3.3 (1.72)	2.16 (1.09)	4.46 (2.50)
Weekly contact, n (%)	2936 (89.46)	11,001 (92.93)	8444 (97.09)
Education, n (%)			
*Primary*	2973 (90.59)	2093 (17.68)	7560 (86.93)
*Secondary*	280 (8.53)	6986 (59.01)	292 (3.36)
*Tertiary*	29 (0.88)	2759 (23.31)	845 (9.72)
Labour force status, n (%)			
*Working*	2163 (65.90)	3448 (29.13)	3345 (38.46)
*Retired*	1049 (31.96)	7169 (60.56)	1042 (11.98)
*Other*	70 (2.13)	1221 (10.31)	4310 (49.56)
Current smoking, n (%)	1084 (33.03)	1462 (12.35)	1044 (12.00)
Weekly alcohol use, n (%)	656 (19.99)	4159 (35.13)	1447 (16.64)
Number of chronic conditions, mean (SD)	0.97 (1.01)	1.82 (1.27)	1.15 (1.06)
ADL disability, n (%)	561 (17.09)	1558 (13.16)	1218 (14.00)
IADL, mean (SD)	0.39 (0.93)	0.15 (0.54)	0.10 (0.41)
Extreme wealth loss, n (%)	530 (16.15)	1183 (9.99)	1477 (16.98)

ADL – activities of daily living, IADL – instrumental activities of daily living, SD – standard deviation

### Extreme wealth loss and subsequent mental well-being

**Figure 1:**
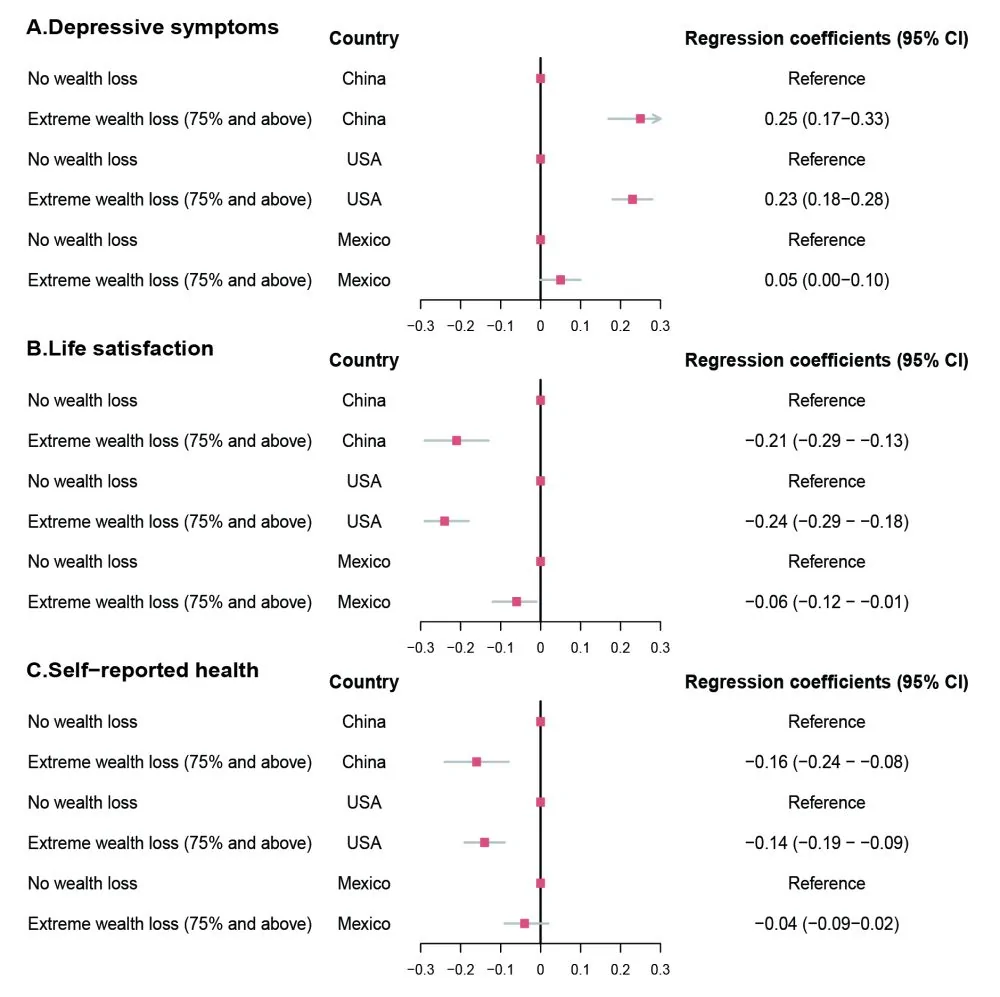
Association of extreme wealth loss with (Panel A) depressive symptoms; (Panel B) life satisfaction; (Panel C) self-reported health. The effect size with 95% CI for each country was separately estimated by the linear mixed model adjusted for age, sex, marital status, household size, contact with others, education, labour force status, current smoking, alcohol consumption, chronic conditions, activities of daily living and instrumental activities of daily living; The higher the scores for the three health outcomes indicate more severe depressive symptoms, greater life satisfaction, and better self-reported health status. CI – confidence interval.

**Figure 2:**
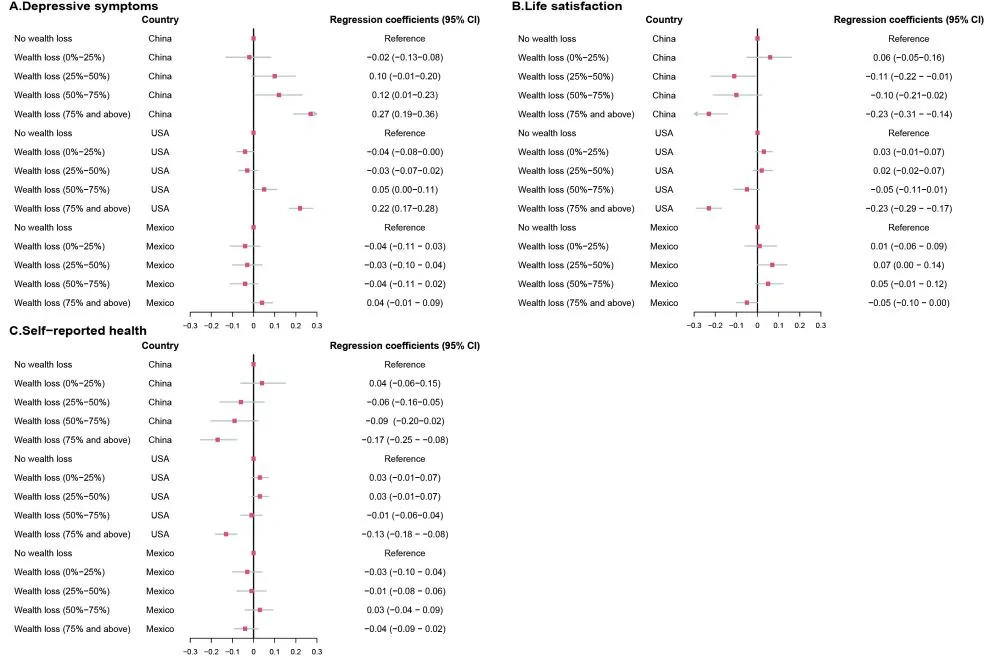
Association of wealth loss gradient with (Panel A) depressive symptoms; (Panel B) life satisfaction; (Panel C) self-reported health. The effect size with 95% CI for each country was separately estimated by the linear mixed model adjusted for age, sex, marital status, household size, contact with others, education, labour force status, current smoking, alcohol consumption, chronic conditions, activities of daily living and instrumental activities of daily living. The higher the scores for the three health outcomes indicate more severe depressive symptoms, greater life satisfaction, and better self-reported health status. CI – confidence interval.

We calculated the associations between extreme wealth loss and subsequent mental well-being (**Figure 1**; Table S4 in the **Online Supplementary Document**). In the fully adjusted models, extreme wealth loss was associated with higher depressive symptoms (OR = 1.3 (95% CI = 1.2–1.4) for China; OR = 1.3 (95% CI = 1.2–1.3) for USA; and OR = 1.1 (95% CI = 1.0–1.1) for Mexico); lower life satisfaction (OR = 0.8 (95% CI = 0.7–0.9) for China; OR = 0.8 (95% CI = 0.7–0.8) for USA; OR = 0.9 (95% CI = 0.9–1.0) for Mexico); and worse self-reported health (OR = 0.9 (95% CI = 0.8–0.9) for China; OR = 0.9 (95% CI = 0.8–0.9) for USA; OR = 1.0 (95% CI = 0.9–1.1) for Mexico). When we further categorised wealth loss into five groups (ranging from no wealth loss to 75% or more), we observed consistent conclusions and found approximate dose-response relationships in both China and the USA (**Figure 2**). Predicted standardised scores for depressive symptoms, life satisfaction and self-reported health showed that the negative association between extreme wealth loss and mental well-being persisted across follow-up waves in China and Mexico, with no statistically significant interaction between exposure group and follow-up time. In USA, we observed a significant interaction between different exposure groups and follow-up time (*P* for interaction _depressive symptoms_<0.01; *P* for interaction _life satisfaction_<0.01; *P* for interaction _self-reported health_<0.01) (Figure S3 in the **Online Supplementary Document**).

### Subgroup analyses and sensitivity analyses

We found that the associations of extreme wealth loss with mental well-being varied among different sub-populations (Figure S4 in the **Online Supplementary Document****)**. All effect sizes for these sub-populations are summarised (Tables S5–7 in the **Online Supplementary Document****)**. When we repeated the analyses using IPTW to address potential confounding caused by time-varying changes in covariates, excluded participants with memory-related and psychological disorders at baseline, created a variable that reflects more nuanced changes in an individual’s relative wealth position within their country over time, or employed inverse probability of censoring weights along with multiple imputation to minimise potential selection bias, our results remained robust (Tables S8–11 in the **Online Supplementary Document**).

## DISCUSSION

In this population-based, cross-national study, we found that extreme wealth loss was associated with subsequent higher depressive symptoms, decreased life satisfaction, and poorer self-reported health among adults aged 50 years and older across three countries. The negative association of extreme wealth loss varied by country and among sub-populations stratified by sociodemographic factors within each country. When we categorised wealth losses into five groups along a gradient, we observed approximate dose-response relationships in both China and USA. Predicted standardised scores for three outcomes showed that the negative association between extreme wealth loss and mental well-being persisted across follow-up waves in China and Mexico, with no statistically significant interaction between wealth loss and follow-up time. In USA, a significant interaction was observed between exposure groups and follow-up time.

Wealth, as a significant social determinant of health, has been extensively studied in recent years, particularly regarding the impact of negative wealth shocks on health outcomes [21,22,27]. Previous research has demonstrated that wealth loss is associated with an increased risk of all-cause mortality [22], decreased cognitive function [21], and psychiatric disorders among older adults [28]. Negative wealth shocks, such as mortgage defaults, investment failures, and retirement, have been significantly linked to deteriorating mental health [13,29,30]. However, existing studies exhibit considerable heterogeneity due to methodological differences, including variations in how mental health is assessed and the selection of instrumental variables for wealth changes. For instance, the impact of retirement on mental health is not solely attributable to a decline in income; it also encompasses changes in social roles and interpersonal networks. Furthermore, assessments of mental well-being often focus on mental disorders, such as depressive symptoms, while neglecting broader dimensions, such as life satisfaction and self-reported health [13,15]. Building on these studies, the value of our research extends beyond merely quantifying wealth and identifying the association between extreme wealth loss and broader measures of mental well-being. It also involves examining cross-country differences in the potential effects of wealth loss on subsequent mental health outcomes.

We found no evidence that the association between extreme wealth loss and mental well-being differed across follow-up waves in China and Mexico, as interaction terms with time were not statistically significant. In contrast, in the USA, the mental health of individuals who experienced extreme wealth loss gradually improved, nearing the levels of the non-wealth loss population during the follow-up period. This difference may be attributed to variations in a country’s macro-social environment and the social safety nets it provides. Middle-income countries, such as China and Mexico, and high-income countries, such as USA, experience different levels of economic inequality and provide varying degrees of social support and security. For instance, while all three countries provide public pensions, the USA allocates a larger share of spending on public pensions [31], and has lower levels of out-of-pocket health expenditure, potentially buffering the longer-term psychological consequences of financial shocks. Such institutional arrangements may enable individuals experiencing financial shocks later in life to recover more quickly. Relevant country-level indicators describing these contextual differences are summarised (Table S8 in the **Online Supplementary Document**). However, because the present analysis includes only three countries, formal statistical evaluation of whether national social protection indicators moderate these associations is not feasible. Future research using larger numbers of countries could formally test these hypotheses using multilevel modelling approaches. Moreover, the observed attenuation over time in the USA may also reflect processes unrelated to macro-social protection, including psychological adaptation following financial shocks, selective attrition of more vulnerable individuals during follow-up, or statistical phenomena such as regression to the mean. In addition, because extreme wealth loss was defined using a relative threshold, comparable proportional losses may correspond to different levels of absolute financial strain across countries with differing wealth distributions. Consequently, although macro-social contexts provide a plausible interpretation of the cross-country patterns observed, these findings should be interpreted cautiously. If causal, our findings further strengthen the arguments that government policies address upstream determinants of disease, with the aim not only of improving the financial situation, but more importantly of providing stable economic security to reduce harm to mental health.

The exact mechanism by which wealth loss leads to mental well-being is unclear, but there are several plausible explanations. The uncertainty associated with living in poverty appears to be a significant contributor to mental health issues. Individuals who experience substantial financial loss may be concerned about their lack of security, including access to adequate social healthcare [5]. This chronic insecurity can result in mental disorders. revealed from the neurological mechanism that the forced loss of social status will generate negative reward prediction error (RPE), and this RPE will strongly activate the lateral habenula (LHb) through the lateral hypothalamus. The activation of LHb cannot only induce depression-like behaviour, but also inhibit the medial prefrontal cortex (mPFC), which is responsible for regulating social competitiveness, and further strengthen the retreat in social competition [4]. In addition, changes in behaviour and lifestyle resulting from negative wealth shocks may also play a significant role. For instance, a sudden decline in wealth can lead to an increase in stress-coping behaviours such as smoking, drinking, and social isolation, whereas an increase in wealth may foster health awareness and protective health behaviours. Variations in these lifestyle changes can result in differing mental health outcomes [32,33].

The main strength of our study is the use of harmonised data from three ageing cohorts. We demonstrate a consistent negative association between extreme wealth loss and mental well-being across different countries, while also observe variations in the potential association of wealth loss with long-term mental health outcomes among these nations. This study had several limitations as well. First, wealth loss was defined over a relatively short observation window between two consecutive survey waves, and it is uncertain whether these changes reflect longer-term wealth trajectories. In addition, because the three longitudinal surveys were conducted in different calendar years, the exposure windows are not identical across countries. However, each exposure window represents wealth change measured between adjacent waves with broadly comparable follow-up intervals (approximately two to three years). Such differences in calendar timing are common in multicohort analyses based on harmonised longitudinal ageing studies and may limit strict cross-country comparability. Second, although we adjusted for as many confounders as possible, residual confounders (such as unmeasured disease) could not be completely eliminated, potentially weakening our results. Third, the impact of reverse causality – where a decline in mental health results in significant wealth loss – cannot be dismissed. However, our findings remain robust even after excluding individuals with memory-related and psychological disorders in our sensitivity analysis. Future research should further explore the potential for a bidirectional relationship between wealth loss and mental well-being.

## CONCLUSIONS

In summary, our findings indicate that extreme wealth loss is associated with poorer subsequent mental well-being. Furthermore, we observed that in the USA, mental health levels among individuals experiencing extreme wealth loss appeared to improve over the follow-up period, whereas similar patterns were not evident in China or Mexico. These cross-national differences may relate to variations in institutional and social protection contexts across countries. From a public health perspective, these findings highlight the importance of identifying older adults who experience substantial financial shocks as a potentially vulnerable group. Further research is needed to better understand how economic and social policies might mitigate the mental health consequences of financial instability in later life.

## Additional material


Online Supplementary Document

## Data Availability

**Data availability:** The original data for this study can be accessed *via* the following websites: the HRS (https://hrs.isr.umich.edu/data-products), CHARLS (https://charls.charlsdata.com/pages/data/111/en.html), and the MHAS (https://www.mhasweb.org/DataProducts/Home.aspx). Harmonized data sets and codebooks for all cohorts are available on the Gateway to Global Aging (https://g2aging.org/hrd/get-data). All data can be accessed after creating an account and providing researcher information.
