# Supplementary material for: Extreme wealth loss and subsequent mental well-being among adults aged ≥50 years: a multicohort study
Source: J Glob Health. 2026 Sep 11;16:04156. doi: 10.7189/jogh.16.04156 (PMC13563152; doi:10.7189/jogh.16.04156)
Supplement: Supplementary file 1 [file jogh-16-04156-s001.pdf]

**Supplement to: Peng K, Xu M, Liu Y, Gong Y. Extreme wealth loss and subsequent mental well-being among adults aged ≥50 years: a multicohort study. J Glob Health. 2026;16:04156.**


Supplementary Methods ....................2

Table S1. Summary of data sources for the outcomes, exposure, and covariates. ....................5

Table S2. The components of wealth variables, by country. ....................6

Table S3. Harmonisation of variables across three longitudinal studies. ....................7

Table S4. Unadjusted associations of extreme wealth loss with mental well-being[a]. ....................8

Table S5. Associations of negative wealth shocks with depressive symptoms, life satisfaction, and self–reported health, categorized by age, sex, marital status, contact with others, education, labor force status in CHARLS (China). ....................10

Table S6. Associations of negative wealth shocks with depressive symptoms, life satisfaction, and self–reported health, categorized by age, sex, marital status, contact with others, education, labor force status in HRS (United States). ....................12

Table S7. Associations of negative wealth shocks with depressive symptoms, life satisfaction, and self–reported health, categorized by age, sex, marital status, contact with others, education, labor force status in MHAS (Mexico). ....................14

Table S8. Country-level indicators of pension generosity, social protection, health financing, and income inequality in China, the United States, and Mexico. ....................16

Table S9. Sensitivity analysis of negative wealth shocks and subsequent mental wellbeing: to address potential confounding due to time-varying changes using inverse probability of treatment weights (IPTW)[a] ....................17

Table S10. Sensitivity analysis of negative wealth shocks and subsequent mental wellbeing: excluding memory-related disease and psychological disorders at baseline[a]. ....................18

Table S11. Sensitivity analysis of negative wealth shocks and subsequent mental wellbeing: a continuous variable was created to represent changes in within-population wealth quintile rankings over the follow-up period ....................19

Table S12. Sensitivity analysis of negative wealth shocks and subsequent mental wellbeing: to account for selection bias resulting from attrition and missing covariates, we created inverse probability weights for attrition and employed multiple imputation using chained equations. ......20

Figure S1. Flowchart of sample selection in the China Health and Retirement Longitudinal Study (CHARLS) (A); Health and Retirement Study (HRS) (B); and Mexican Health and Aging Study (MHAS) (C).. ....................21

Figure S2. Directed acyclic graph (DAG) that guided selection of plausible confounders. ...........22

**Figure** S3. Predicted standardised scores for depressive symptoms, life satisfaction, and self-reported health over time by extreme wealth loss. .................... 23

Figure S4. Associations of extreme wealth loss with (A) depressive symptoms; (B) life satisfaction; (C) self-reported health in different subpopulations. .................... 24

| JoGH guideline item | Purpose |
|---|---|
| 1. Please list all papers published by each co-author in previous three years that were based on secondary analysis of a big data repository | The authors have reviewed their publications in the past three years.<br>*Lifestyle factors in the association of shift work and depression and anxiety. JAMA Network Open. 2023.*<br>*Association of premature natural and surgical menopause with incidence of depression requiring hospitalization: a prospective cohort study. American Journal of Obstetrics and Gynecology. 2025.*<br>*Total and regional fat-to-muscle mass ratio in relation to all-cause and cause-specific mortality in men and women. Journal of Clinical Endocrinology & Metabolism. 2025.*<br>*Engagement in leisure activities and cognitive function by socioeconomic groups in China: a prospective cohort study.Journal of Affective Disorders. 2023.*<br>*Association of Frailty With Risk of Incident Hospital-Treated Infections in Middle-Aged and Older Adults: A Large-Scale Prospective Cohort Study.The journals of gerontology.2024 .*<br>None of these publications duplicate the present research question, exposure definition, analytical design, or conclusions. |
| 2. Please explain the key elements of your study design and the use of the available datasets that make your study an original scientific contribution | This study provides an original contribution by examining extreme wealth loss as the main exposure and subsequent mental well-being as the outcome in a harmonised longitudinal multicohort design. The study focuses on within-person wealth change rather than static socioeconomic position, and evaluates temporal associations using longitudinal follow-up. |
| 3. Please list all publications that addressed similar research questions in the same dataset and indicate where you cited them in your paper | We reviewed previous studies using the same or related ageing cohort datasets and cited relevant work in the Introduction and Discussion. To our knowledge, no previous study has addressed the same research question using the same exposure definition, outcome framework, and multicohort longitudinal design. |

| JoGH guideline item | Purpose |
| --- | --- |
| 4. Please explain how you addressed multiple testing through an appropriately rigorous statistical threshold and indicate this in the methods section | The primary exposure, outcomes, and modelling strategy were specified a priori. Sensitivity and subgroup analyses were conducted to assess robustness and potential heterogeneity and were interpreted as secondary or exploratory. We avoided overinterpretation of isolated statistically significant findings and based conclusions primarily on the consistency, magnitude, and direction of the main results. |
| 5. Please declare to what extent have AI chatbots been used in developing your paper and to which parts of the paper did they contribute | No AI tools were used for data management, statistical analysis, generation of results, or interpretation of findings. If language-editing tools were used, their role was limited to improving clarity and grammar. All manuscript content, analyses, interpretations, and conclusions were reviewed and approved by the authors. |

## Supplementary Methods

### Linear mixed model

We employed linear mixed models with years since baseline as the timescale and person-specific random intercepts to estimate the association between negative wealth shocks and subsequent mental well-being within each country. The linear mixed model is a longitudinal data approach that not only accounts for the correlation between repeated measures on the same individual but also effectively handles missing data and unbalanced designs over the follow-up period. The basic model included extreme wealth loss, years since baseline, and their interaction. All models were subsequently adjusted for age, sex, marital status, household size, contact with others, education, labour force status, current smoking, alcohol consumption, chronic conditions, ADL and instrumental activities of daily living (IADL).

The linear mixed model can be expressed as equation:

$$y_{ij} = \beta_0 + \beta_1 \text{ extreme wealth loss}_i + \beta_2 \text{ Time}_{ij} + \beta_3 \text{ extreme wealth loss}_i \times \text{Time}_{ij} + X_i \boldsymbol{\beta}' + b_{0i} + \epsilon_{ij},\ i=1, \dots, N;\ j = 1, \dots, n_i$$

where $y_{ij}$ is the mental health outcome for the $i$th individual measured at the $j$th follow-up, extreme wealth loss$_i$ denotes the wealth loss information at the first two waves, Time$_{ij}$ is the year since baseline, $X_i$ is the $N \times p$ design matrix for baseline covariates, $\boldsymbol{\beta}'$ is a $p \times 1$ vector of coefficients, $b_{0i} \sim N(0, \sigma_b^2)$ is the random effect for the $i$th individual and $\epsilon_{ij} \sim N(0, \sigma^2)$ is the random error.

### Inverse probability weights of attrition

To account for selection bias due to attrition, we created the stabilized inverse probability weights of attrition using the following equation:

$$IPW_{ij} = \prod_{k=0}^{j} \frac{\Pr\{C_{ik} = 0 | C_{i(k-1)} = 0, X_i, V_i\}}{\Pr\{C_{ik} = 0 | C_{i(k-1)} = 0, X_i, \bar{L}_{i(k-1)}\}}$$

where $C_{ik} = 0$ indicates that participant $i$ is not censored by wave $k$; $X_i$ represents the exposure; $V_i$ represents baseline covariates in our study; $\bar{L}_{i(k-1)}$ represents time-varying variables at the previous wave (i.e., wave $k$ - 1), including standardized values for depressive symptoms, life satisfaction, and self-reported health. The numerator represents the conditional probability of remaining uncensored up to wave $k$, given the baseline covariates and exposure. The denominator represents the conditional probability of remaining uncensored up to wave $k$, given the baseline covariates, exposure, and standardized scores for depressive symptoms, life satisfaction, and self-reported health at the previous wave. We estimated these probabilities using logistic regression models. The weights for each participant at each wave were proportional to the inverse of the wave-specific probability of remaining uncensored. Since there were only two waves of follow-up in Mexico, we estimated the inverse probability weights of attrition as follows:

$$IPW_i = \frac{1}{\Pr\{C_i = 0 | X_i, V_i\}}$$

**Table S1.** Summary of data sources for the outcomes, exposure, and covariates.

| Country | Data sources | | |
|---|---|---|---|
| | **Exposure: negative wealth shocks (time points of data collection)** | **Covariates (time point)** | **Outcomes: depressive symptoms, life satisfaction, Self-reported health (time point)** |
| China | CHARLS(wave 1-2) | CHARLS(wave 1) | CHARLS(wave 2-4) |
| United States | HRS(wave 9-10) | HRS(wave 9) | HRS(wave 10-14) |
| Mexico | MHAS(wave 3-4) | MHAS(wave 3) | MHAS(wave 4-5) |

**Table S2.** The components of wealth variables, by country.

| Components | China | United States | Mexico |
|---|---|---|---|
| Primary residence and other real estate | Yes[b] | Yes | Yes |
| Transportation | Yes[c] | Yes | Yes |
| Business | Yes[d] | Yes | Yes |
| Non-housing financial wealth[a] | Yes | Yes | Yes |
| Debts | Yes | Yes | Yes |

[a] Non-housing financial wealth includes stocks, mutual funds, investment trusts, checking accounts, savings accounts, and money market accounts, CDs, government savings bonds, treasury bills, bonds, bond funds and all other savings

[b] The value of primary residence with the percentage of ownership are considered

[c] The total value of transportation vehicles is measured at the household level at wave 1-2.

[d] The total value of non-financial assets, fixed capital assets, irrigable land, and all livestock and aquatic life is included and measured at the household level at wave 1-2.

**Table S3.** Harmonisation of variables across three longitudinal studies.

| Variable | Harmonized value | Measurements in three studies | | |
|---|---|---|---|---|
| | | CHARLS | HRS | MHAS |
| Depressive symptoms | Continuous | Center for Epidemiologic Studies Depression Scale (CES-D) [10 items] (ranging 0–30) | CES-D [8 items] (ranging 0– 8) | CES-D [9 items] (ranging 0–9) |
| Life satisfaction | Continuous | How satisfied are you with your life-as-a-whole?(5-point scale) | How satisfied are you with your life as a whole? (5-point scale) | I am satisfied with life. (3-point scale) |
| Self-reported health | Continuous | Would you say your health is very good, good, fair, poor or very poor? (reversely coded] | Would you say your health is excellent, very good, good, fair, or poor? (reversely coded) | Would you say your health is excellent, very good, good, fair, or poor? (reversely coded) |
| Age, years | Continuous | The respondent's age | | |
| Sex | Male<br>Female | The respondent's sex | | |
| Marital status | Married<br>Unmarried | Married or partnered<br>Separated, divorced, widowed, or never married | | |
| Household size | Continuous | Number of people living in the household | | |
| Contact with others | Weekly contact | Whether the respondent contacts with any of their parents in person,or children either in person, by mail or e- mail at least once a week?<br>• Yes | Whether the respondent contacts with any of their children, parents, relatives, and friends either in person or by phone, mail, or e-mail at least once a week in the past year?<br>• Yes | Whether the respondent contacts with any of their children, relatives, and friends, either in person, by mail, or by telephone at least once a week?<br>• Yes |
| | Less than weekly contact | • No | • No | • No |
| Education | Primary<br>Secondary<br>Tertiary | Less than upper secondary education<br>Upper secondary & vocational training<br>Tertiary education | | |
| Labor force status | Working | Agricultural employed, agricultural agricultural agricultural self-employed, non-employed, non-self-employed, or non-agricultural unpaid family business | Working full-time or part-time | Working |
| | Retired | Retired | Partly retired or retired | Retired |
| | Others | Unemployed or never worked | Unemployed, disabled, or not in the labor force | Unemployed, disabled, or not in the labor force |

| | | | | |
|---|---|---|---|---|
| Current smoking | Yes<br>No | Whether the respondent smokes now. | | |
| Alcohol consumption | Less than weekly alcohol use | The highest frequency of drinking behavior during the last year that the respondent reports for any one of the three types of alcohol (i.e., white liquor, whisky, and others; beer; wine or rice wine).<br>• Less than once a month<br>• Once a month<br>• Two to three days a month<br>• Once or twice a month | In the last three months, on average, how many days per week have you had any alcohol to drink?<br>• 0 day/week | The number of days per week the respondent drinks.<br>• 0 day/week |
| | Weekly alcohol use | • Once a week<br>• Daily<br>• Twice a day<br>• More than twice a day | • 1–7 day/week | • 1–7 day/week |
| Number of chronic conditions | Continuous | Total number of chronic conditions, including hypertension, diabetes, cancer, chronic lung disease, heart problems, stroke, and arthritis. All these chronic conditions were ascertained through self-reported physicians' diagnoses. | | |
| ADL | Yes | Difficulties with any activities of daily living (ADLs), including dressing, bathing,eating, getting in and out of bed, using the toilet, and controlling urination and defecation. | Difficulties with any activities of daily living (ADLs), including dressing, bathing, eating, getting in and out of bed, using the toilet, and walking across a room. | Difficulties with any activities of daily living (ADLs), including dressing, bathing, eating, getting in and out of bed, using the toilet, and walking across a room. |
| | No | No difficulty with all ADLs. | | |
| IADL | Continuous | Number of limitations on IADLs, including managing money, taking medications, shopping for groceries, preparing meals, and cleaning house. (0–5) | Number of limitations on instrumental activities of daily living (IADLs), including managing money, taking medications, shopping for groceries, preparing meals, and using the phone. (0–5) | Number of limitations on IADLs, including managing money, taking medications, shopping for groceries, and preparing meals. (0–4) |

**Supplemental Table 4.** Unadjusted associations of extreme wealth loss with mental well-being[a].

| Country | Exposure | Depressive | Life satisfaction | Self−reported health |
|---|---|---|---|---|

| | | symptoms | | |
|---|---|---|---|---|
| China | No extreme wealth loss | Reference | Reference | Reference |
| | Extreme wealth loss | 0.27 (0.18-0.36) | -0.21 (-0.29 - -0.12) | -0.18 (-0.27 - -0.10) |
| United States | No extreme wealth loss | Reference | Reference | Reference |
| | Extreme wealth loss | 0.41 (0.36-0.47) | -0.36 (-0.41 - -0.30) | -0.35 (-0.40 - -0.29) |
| Mexico | No extreme wealth loss | Reference | Reference | Reference |
| | Extreme wealth loss | 0.09 (0.04-0.15) | -0.08 (-0.13 - -0.02) | -0.07 (-0.13 - -0.02) |

[a]The effect size with 95% CI for each country was separately estimated by the linear mixed model; The higher the scores for the three health outcomes indicate more severe depressive symptoms, greater life satisfaction, and better self-reported health status.

**Table S5.** Associations of negative wealth shocks with depressive symptoms, life satisfaction, and self−reported health, categorized by age, sex, marital status, contact with others, education, labor force status in CHARLS (China).

| Subgroup analyses[a] | |
|---|---|
| **By age** | |
| **Age 50-64** | **≥ Age 65** |
| **Depressive symptoms** | **Depressive symptoms** |
| 0.25 (0.15-0.35) | 0.26 (0.11-0.42) |
| **Life satisfaction** | **Life satisfaction** |
| -0.24 (-0.34 - -0.14) | -0.15 (-0.31 - 0.00) |
| **Self−reported health** | **Self−reported health** |
| -0.18 (-0.28 - -0.09) | -0.11 (-0.27 - 0.04) |
| **By sex** | |
| **Male** | **Female** |
| **Depressive symptoms** | **Depressive symptoms** |
| 0.27 (0.15-0.39) | 0.24 (0.12-0.36) |
| **Life satisfaction** | **Life satisfaction** |
| -0.18 (-0.29 - -0.06) | -0.25 (-0.37 - -0.12) |
| **Self−reported health** | **Self−reported health** |
| -0.16 (-0.27 - -0.05) | -0.16 (-0.28 - -0.04) |
| **By marital status** | |
| **Unmarried** | **Married** |
| **Depressive symptoms** | **Depressive symptoms** |
| 0.38 (0.14-0.62) | 0.23 (0.14-0.32) |
| **Life satisfaction** | **Life satisfaction** |
| -0.33 (-0.57 - -0.09) | -0.19 (-0.28 - -0.10) |
| **Self−reported health** | **Self−reported health** |
| -0.13 (-0.37 - 0.11) | -0.16 (-0.25 - -0.08) |
| **By contact** | |
| **≥ weekly** | **< weekly** |
| **Depressive symptoms** | **Depressive symptoms** |
| 0.26 (0.17-0.34) | 0.21 (-0.03 - 0.46) |
| **Life satisfaction** | **Life satisfaction** |
| -0.21 (-0.30 - -0.12) | -0.24 (-0.50 - 0.01) |
| **Self−reported health** | **Self−reported health** |
| -0.14 (-0.23 - -0.06) | -0.24 (-0.48 - 0.01) |
| **By education** | |
| **Primary** | **Secondary or tertiary** |
| **Depressive symptoms** | **Depressive symptoms** |
| 0.26 (0.17-0.35) | 0.13 (-0.18 - 0.44) |
| **Life satisfaction** | **Life satisfaction** |
| -0.22 (-0.31 - -0.14) | -0.03 (-0.33 - 0.28) |
| **Self−reported health** | **Self−reported health** |

| | |
|---|---|
| -0.17 (-0.25 - -0.08) | -0.07 (-0.37 - 0.22) |
| **By labor force status** | |
| **Working** | **Retired / Others** |
| **Depressive symptoms** | **Depressive symptoms** |
| 0.11 (0.00-0.21) | 0.49 (0.36-0.63) |
| **Life satisfaction** | **Life satisfaction** |
| -0.12 (-0.23 - -0.02) | -0.35 (-0.49 - -0.21) |
| **Self−reported health** | **Self−reported health** |
| -0.10 (-0.20 - 0.00) | -0.25 (-0.38 - -0.11) |

[a]The effect size with 95% CI for each subgroup was separately estimated by the linear mixed model adjusted for age, sex, marital status, household size, contact with others, education, labour force status, current smoking, alcohol consumption, chronic conditions, ADL and instrumental activities of daily living (IADL), excluding the variable itself; The higher the scores for the three health outcomes indicate more severe depressive symptoms, greater life satisfaction, and better self-reported health status.

**Table S6.** Associations of negative wealth shocks with depressive symptoms, life satisfaction, and self−reported health, categorized by age, sex, marital status, contact with others, education, labor force status in HRS (United States).

| **Subgroup analyses[a]** | |
|---|---|
| **By age** | |
| **Age 50-64** | **≥ Age 65** |
| **Depressive symptoms** | **Depressive symptoms** |
| 0.21 (0.14-0.29) | 0.22 (0.15-0.29) |
| **Life satisfaction** | **Life satisfaction** |
| -0.28 (-0.36 - -0.20) | -0.19 (-0.26 - -0.11) |
| **Self−reported health** | **Self−reported health** |
| -0.16 (-0.23 - -0.09) | -0.11 (-0.18 - -0.04) |
| **By sex** | |
| **Male** | **Female** |
| **Depressive symptoms** | **Depressive symptoms** |
| 0.33 (0.24-0.42) | 0.18 (0.12-0.24) |
| **Life satisfaction** | **Life satisfaction** |
| -0.29 (-0.38 - -0.20) | -0.20 (-0.27 - -0.13) |
| **Self−reported health** | **Self−reported health** |
| -0.18 (-0.27 - -0.10) | -0.12 (-0.18 - -0.06) |
| **By marital status** | |
| **Unmarried** | **Married** |
| **Depressive symptoms** | **Depressive symptoms** |
| 0.16 (0.08-0.24) | 0.29 (0.22-0.36) |
| **Life satisfaction** | **Life satisfaction** |
| -0.14 (-0.22 - -0.06) | -0.32 (-0.39 - -0.25) |
| **Self−reported health** | **Self−reported health** |
| -0.09 (-0.16 - -0.02) | -0.18 (-0.25 - -0.12) |
| **By contact** | |
| **≥ weekly** | **< weekly** |
| **Depressive symptoms** | **Depressive symptoms** |
| 0.24 (0.18-0.29) | 0.15 (-0.03 - 0.34) |
| **Life satisfaction** | **Life satisfaction** |
| -0.24 (-0.30 - -0.19) | -0.17 (-0.37 - 0.02) |
| **Self−reported health** | **Self−reported health** |
| -0.14 (-0.19 - -0.09) | -0.13 (-0.31 - 0.04) |
| **By education** | |
| **Primary** | **Secondary or tertiary** |
| **Depressive symptoms** | **Depressive symptoms** |
| 0.20 (0.11-0.30) | 0.25 (0.19-0.31) |
| **Life satisfaction** | **Life satisfaction** |
| -0.16 (-0.26 - -0.06) | -0.27 (-0.34 - -0.21) |
| **Self−reported health** | **Self−reported health** |

| | |
|---|---|
| -0.16 (-0.26 - -0.06) | -0.15 (-0.21 - -0.09) |
| **By labor force status** | |
| **Working** | **Retired / Others** |
| **Depressive symptoms** | **Depressive symptoms** |
| 0.33 (0.23-0.42) | 0.20 (0.13-0.26) |
| **Life satisfaction** | **Life satisfaction** |
| -0.36 (-0.45 - -0.26) | -0.19 (-0.25 - -0.12) |
| **Self−reported health** | **Self−reported health** |
| -0.17 (-0.26 - -0.08) | -0.13 (-0.19 - -0.07) |

[a]The effect size with 95% CI for each subgroup was separately estimated by the linear mixed model adjusted for age, sex, marital status, household size, contact with others, education, labour force status, current smoking, alcohol consumption, chronic conditions, ADL and instrumental activities of daily living (IADL), excluding the variable itself; The higher the scores for the three health outcomes indicate more severe depressive symptoms, greater life satisfaction, and better self-reported health status.

**Table S7.** Associations of negative wealth shocks with depressive symptoms, life satisfaction, and self–reported health, categorized by age, sex, marital status, contact with others, education, labor force status in MHAS (Mexico).

| Subgroup analyses[a] | |
|---|---|
| **By age** | |
| **Age 50-64** | **≥ Age 65** |
| **Depressive symptoms** | **Depressive symptoms** |
| 0.12 (0.05-0.19) | -0.04 (-0.12 - 0.04) |
| **Life satisfaction** | **Life satisfaction** |
| -0.10 (-0.18 - -0.03) | 0.01 (-0.08 - 0.09) |
| **Self−reported health** | **Self−reported health** |
| -0.05 (-0.12 - 0.01) | -0.01 (-0.09 - 0.07) |
| **By sex** | |
| **Male** | **Female** |
| **Depressive symptoms** | **Depressive symptoms** |
| 0.07 (-0.01 - 0.16) | 0.04 (-0.03 - 0.11) |
| **Life satisfaction** | **Life satisfaction** |
| -0.04 (-0.13 - 0.05) | -0.07 (-0.14 - 0.00) |
| **Self−reported health** | **Self−reported health** |
| -0.05 (-0.14 - 0.03) | -0.02 (-0.09 - 0.04) |
| **By marital status** | |
| **Unmarried** | **Married** |
| **Depressive symptoms** | **Depressive symptoms** |
| 0.00 (-0.10 - 0.09) | 0.08 (0.02-0.15) |
| **Life satisfaction** | **Life satisfaction** |
| -0.02 (-0.12 - 0.07) | -0.08 (-0.15 - -0.01) |
| **Self−reported health** | **Self−reported health** |
| 0.01 (-0.08 - 0.11) | -0.06 (-0.12 - 0.00) |
| **By contact** | |
| **≥ weekly** | **< weekly** |
| **Depressive symptoms** | **Depressive symptoms** |
| 0.05 (-0.01 - 0.10) | 0.16 (-0.11 - 0.43) |
| **Life satisfaction** | **Life satisfaction** |
| -0.06 (-0.12 - 0.00) | -0.05 (-0.33 - 0.23) |
| **Self−reported health** | **Self−reported health** |
| -0.04 (-0.09 - 0.02) | 0.00 (-0.28 - 0.27) |
| **By education** | |
| **Primary** | **Secondary or tertiary** |
| **Depressive symptoms** | **Depressive symptoms** |
| 0.05 (-0.01 - 0.11) | 0.06 (-0.10 - 0.22) |
| **Life satisfaction** | **Life satisfaction** |
| -0.07 (-0.13 - -0.01) | 0.02 (-0.15 - 0.18) |
| **Self−reported health** | **Self−reported health** |

| | |
|---|---|
| -0.02 (-0.08 - 0.03) | -0.11 (-0.27 - 0.05) |
| **By labor force status** | |
| **Working** | **Retired / Others** |
| **Depressive symptoms** | **Depressive symptoms** |
| 0.14 (0.05-0.22) | 0.00 (-0.06 - 0.07) |
| **Life satisfaction** | **Life satisfaction** |
| -0.04 (-0.13 - 0.05) | -0.07 (-0.14 - 0.00) |
| **Self−reported health** | **Self−reported health** |
| -0.05 (-0.13 - 0.03) | -0.03 (-0.10 - 0.04) |

[a]The effect size with 95% CI for each subgroup was separately estimated by the linear mixed model adjusted for age, sex, marital status, household size, contact with others, education, labour force status, current smoking, alcohol consumption, chronic conditions, ADL and instrumental activities of daily living (IADL), excluding the variable itself; The higher the scores for the three health outcomes indicate more severe depressive symptoms, greater life satisfaction, and better self-reported health status.

**Table S8.** Country-level indicators of pension generosity, social protection, health financing, and income inequality in China, the United States, and Mexico.

| Country | Net pension replacement rate (100% average wage)[a] | Net social expenditure (% GDP)[b] | Out-of-pocket health expenditure (% of total health expenditure)[c] | Universal health coverage (UHC service coverage index)[d] | Gini index [e] |
|---|---|---|---|---|---|
| China | 45.1 | 10.9 | 32.17 | 79.5 | 36.0 |
| United States | 51.3 | 33.2 | 10.93 | 88.0 | 41.8 |
| Mexico | 79.6 | 9.4 | 41.24 | 75.6 | 43.5 |

[a] Net pension replacement rate at 100% of average wage. Data for China were obtained from the China Pension Finance Development Report. Data for the United States and Mexico were obtained from the Organisation for Economic Co-operation and Development Pensions at a Glance database.

[b] Net social expenditure as a percentage of gross domestic product (GDP). Data for China were obtained from the China Finance Statistical Yearbook and the official website of the Ministry of Finance. Data for the United States and Mexico were obtained from the Organisation for Economic Co-operation and Development Social Expenditure Database (SOCX).

[c] Out-of-pocket health expenditure as a percentage of total health expenditure. Data were obtained from the World Health Organization Global Health Expenditure Database (updated December 2025).

[d] Universal Health Coverage (UHC) service coverage index. Data were obtained from the World Health Organization.

[e] Gini index measuring income inequality. Data were obtained from the World Bank World Development Indicators database.

All indicators represent the most recent available data around 2021–2023.

**Table S9.** Sensitivity analysis of negative wealth shocks and subsequent mental wellbeing: to address potential confounding due to time-varying changes using inverse probability of treatment weights (IPTW)[a].

| **Country** | **Exposure** | **Depressive symptoms**[b] | **Life satisfaction**[b] | **Self−reported health**[b] |
|---|---|---|---|---|
| China | No extreme wealth loss | Reference | Reference | Reference |
| | Extreme wealth loss | 0.25 (0.17-0.34) | -0.22 (-0.31 - -0.13) | -0.17 (-0.25 - -0.09) |
| United States | No extreme wealth loss | Reference | Reference | Reference |
| | Extreme wealth loss | 0.21 (0.16-0.26) | -0.23 (-0.29 - -0.18) | -0.12 (-0.17 - -0.07) |
| Mexico | No extreme wealth loss | Reference | Reference | Reference |
| | Extreme wealth loss | 0.06 (0.00-0.11) | -0.07 (-0.12 - -0.01) | -0.04 (-0.09 - 0.00) |

[a]IPTW involved calculating the probability of each participant experiencing a extreme wealth loss based on observed covariates, and then using these probabilities to create a weighted sample in which the distribution of covariates is independent of the exposure.The weights were derived from a logistic regression model that included baseline covariates. The application of IPTW mitigated confounding bias by balancing covariates across exposure groups to obtain unbiased estimates.

**[b]**The effect size with 95% CI for each country was separately estimated by the linear mixed model adjusted for age, sex, marital status, household size, contact with others, education, labour force status, current smoking, alcohol consumption, chronic conditions, ADL and instrumental activities of daily living (IADL); The higher the scores for the three health outcomes indicate more severe depressive symptoms, greater life satisfaction, and better self-reported health status.

**Table S10.** Sensitivity analysis of negative wealth shocks and subsequent mental wellbeing: excluding memory-related disease and psychological disorders at baseline[a].

| Country | Exposure | Depressive symptoms[b] | Life satisfaction[b] | Self−reported health[b] |
|---|---|---|---|---|
| China | No extreme wealth loss | Reference | Reference | Reference |
| | Extreme wealth loss | 0.25 (0.16-0.33) | -0.20 (-0.28 - -0.11) | -0.16 (-0.25 - -0.08) |
| United States | No extreme wealth loss | Reference | Reference | Reference |
| | Extreme wealth loss | 0.23 (0.17-0.29) | -0.24 (-0.30 - -0.18) | -0.12 (-0.18 - -0.07) |

[a]Memory-related diseases were Alzheimer's disease, dementia, Parkinson's disease, and other memory-related conditions. Psychological disorders were emotional, nervous, and psychiatric problems. Memory-related diseases and psychological disorders were not available in MHAS.

[b]The effect size with 95% CI for each country was separately estimated by the linear mixed model adjusted for age, sex, marital status, household size, contact with others, education, labour force status, current smoking, alcohol consumption, chronic conditions, ADL and instrumental activities of daily living (IADL); The higher the scores for the three health outcomes indicate more severe depressive symptoms, greater life satisfaction, and better self-reported health status.

**Table S11.** Sensitivity analysis of negative wealth shocks and subsequent mental wellbeing: a continuous variable was created to represent changes in within-population wealth quintile rankings over the follow-up period, capturing more subtle fluctuations in relative wealth rankings over time[a].

| Country | Exposure | Depressive symptoms[b] | Life satisfaction[b] | Self−reported health[b] |
|---|---|---|---|---|
| China | No extreme wealth loss | Reference | Reference | Reference |
| | Extreme wealth loss | -0.03 (-0.05 - -0.01) | 0.04 (0.02-0.06) | 0.02 (0.00-0.04) |
| United States | No extreme wealth loss | Reference | Reference | Reference |
| | Extreme wealth loss | -0.03 (-0.05 - -0.01) | 0.04 (0.02-0.06) | 0.00 (-0.02-0.02) |
| Mexico | No extreme wealth loss | Reference | Reference | Reference |
| | Extreme wealth loss | 0.01 (-0.01 - 0.02) | 0.00 (-0.01 - 0.01) | 0.00 (-0.01 - 0.02) |

[a]A continuous variable was created to represent changes in within-population wealth quintile rankings over the follow-up period, with a range from -4 to 4. This variable captures more subtle fluctuations in an individual's relative wealth ranking within their country over time. For instance, moving from the top 20% of the population wealth distribution to the bottom 20% would yield a value of -4 (i.e., a decline of four quintiles). Here, -4 indicates the maximum loss of wealth gradient, while 4 signifies the maximum gain of wealth gradient.

[b]The effect size with 95% CI for each country was separately estimated by the linear mixed model adjusted for age, sex, marital status, household size, contact with others, education, labour force status, current smoking, alcohol consumption, chronic conditions, ADL and instrumental activities of daily living (IADL); The higher the scores for the three health outcomes indicate more severe depressive symptoms, greater life satisfaction, and better self-reported health status.

**Table S12.** Sensitivity analysis of negative wealth shocks and subsequent mental wellbeing: to account for selection bias resulting from attrition and missing covariates, we created inverse probability weights for attrition and employed multiple imputation using chained equations.

| Country | Exposure | Depressive symptoms[a] | Life satisfaction[a] | Self−reported health[a] |
|---|---|---|---|---|
| China | No extreme wealth loss | Reference | Reference | Reference |
| | Extreme wealth loss | 0.20 (0.14-0.27) | -0.14 (-0.21 - -0.07) | -0.09 (-0.16 - -0.02) |
| United States | No extreme wealth loss | Reference | Reference | Reference |
| | Extreme wealth loss | 0.18 (0.13-0.22) | -0.20 (-0.25 - -0.15) | -0.12 (-0.17 - -0.08) |
| Mexico | No extreme wealth loss | Reference | Reference | Reference |
| | Extreme wealth loss | 0.03 (-0.01-0.07) | -0.06 (-0.11 - -0.01) | -0.01 (-0.06 - 0.03) |

[a]The effect size with 95% CI for each country was separately estimated by the linear mixed model adjusted for age, sex, marital status, household size, contact with others, education, labour force status, current smoking, alcohol consumption, chronic conditions, ADL and instrumental activities of daily living (IADL); The higher the scores for the three health outcomes indicate more severe depressive symptoms, greater life satisfaction, and better self-reported health status.

**Figure S1.** Flowchart of sample selection in the China Health and Retirement Longitudinal Study (CHARLS) (A); Health and Retirement Study (HRS) (B); and Mexican Health and Aging Study (MHAS) (C). In the present study, outcomes included depressive symptoms, life satisfaction, and self-reported health.

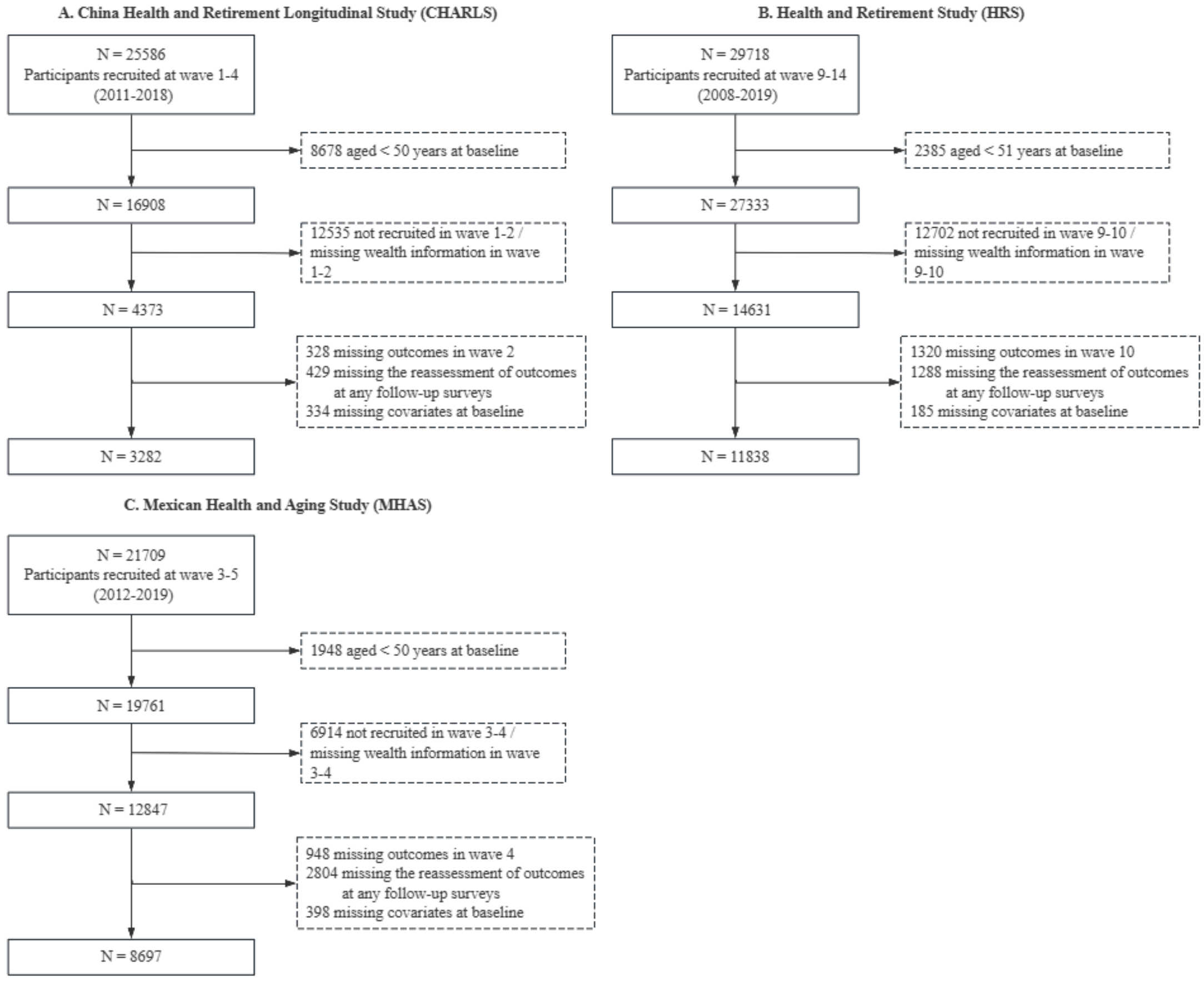

**Figure S2.** Directed acyclic graph (DAG) that guided selection of plausible confounders.

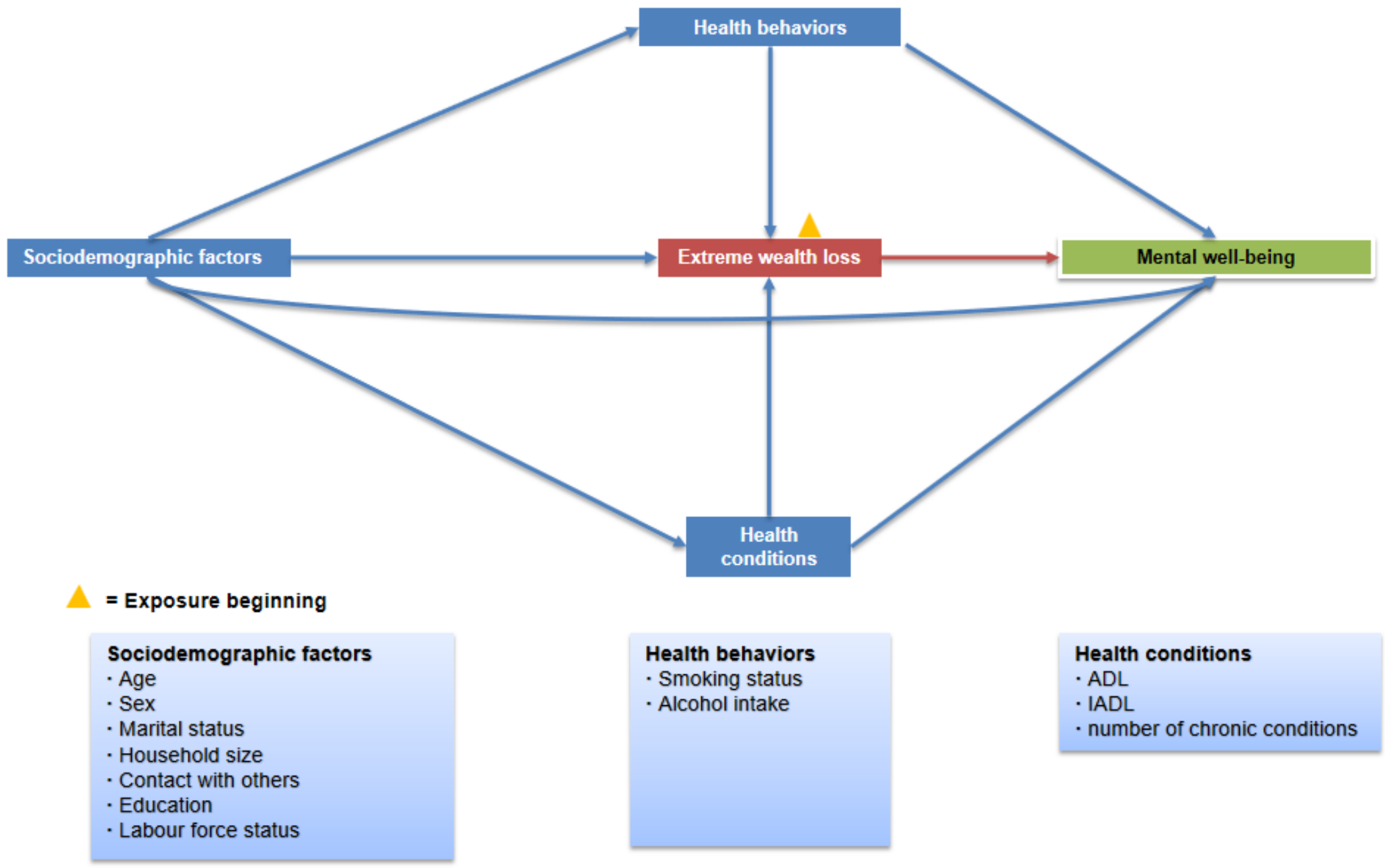


Note: The red, green, and blue nodes respectively represent the exposure measured from the baseline (T0) of the study periods to the follow-up time-point (T1), the outcomes measured at the follow-up time ($T1$-$T_X$), and the confounders of the sociodemographic, lifestyle-related, and health-related factors measured at the baseline (T0).

**Figure S3.** Predicted standardised scores for depressive symptoms, life satisfaction, and self-reported health over time by extreme wealth loss.

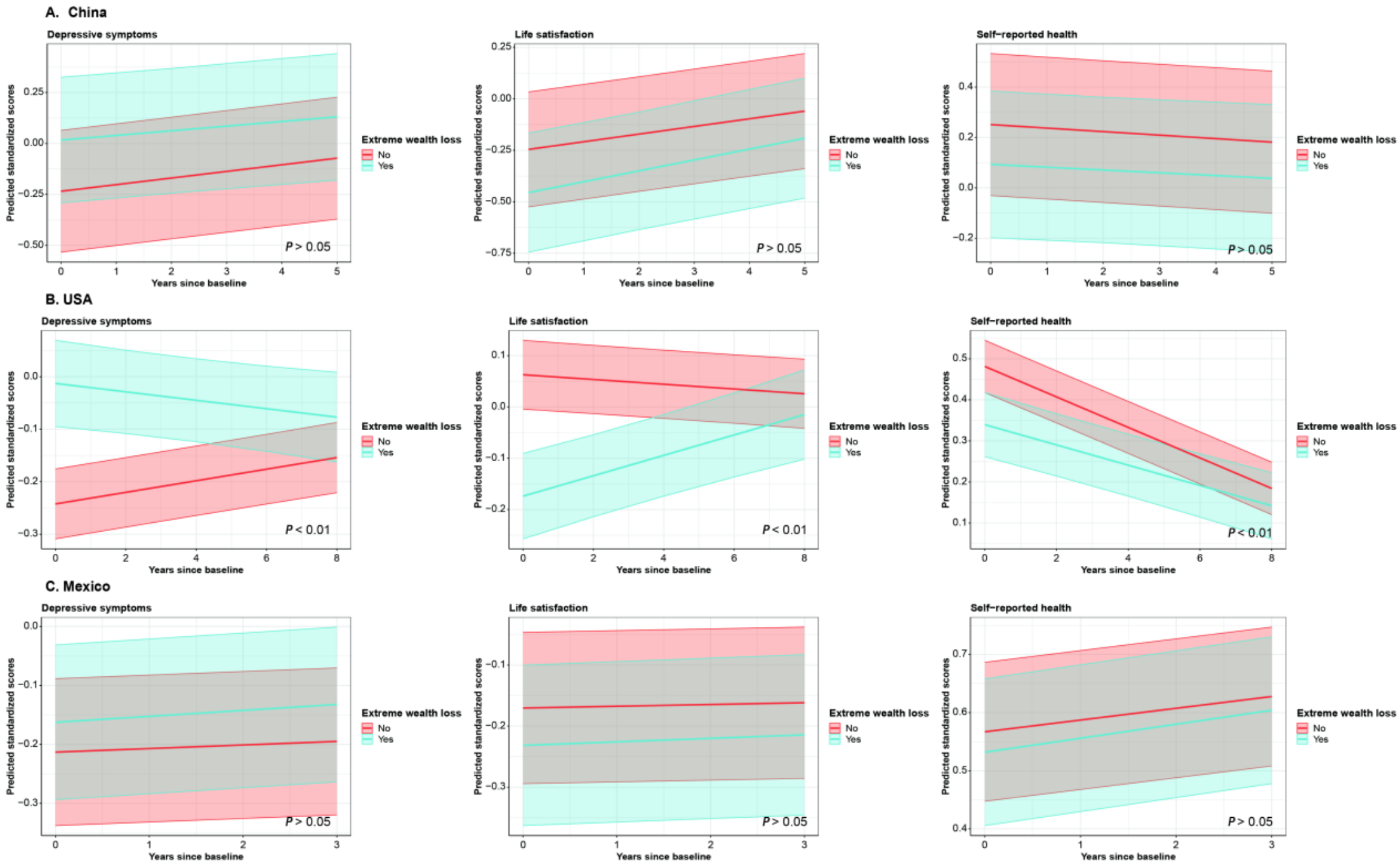


The values with 95% CI for each country was separately estimated by the linear mixed model adjusted for age, sex, marital status, household size, contact with others, education, labour force status, current smoking, alcohol consumption, chronic conditions, activities of daily living and instrumental activities of daily living. The higher the scores for the three health outcomes indicate more severe depressive symptoms, greater life satisfaction, and better self-reported health status. CI – confidence interval.

**Figure S4.** Associations of extreme wealth loss with (A) depressive symptoms; (B) life satisfaction; (C) self-reported health in different subpopulations.

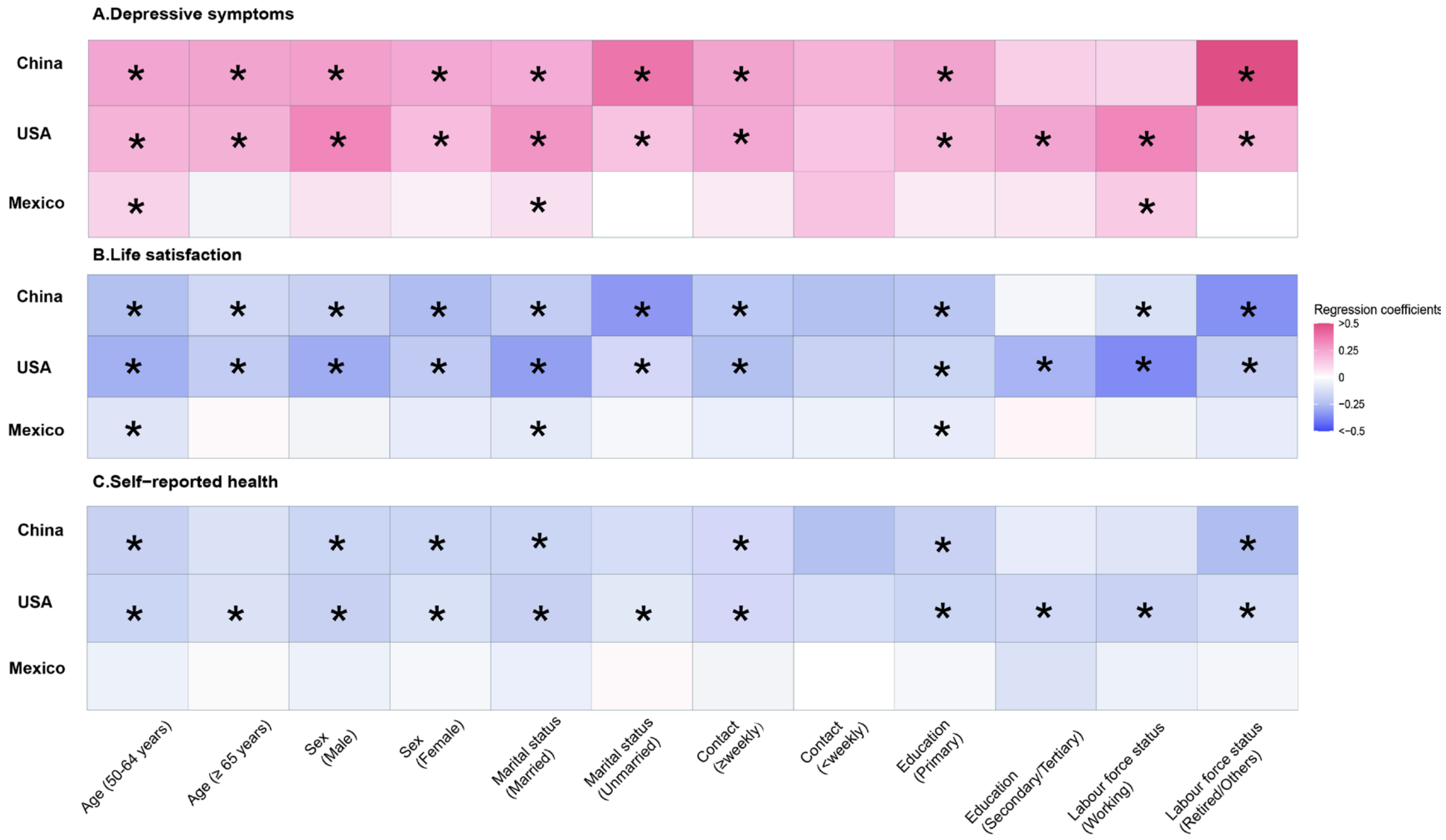


The effect size with 95% CI for each subgroup was separately estimated by the linear mixed model adjusted for age, sex, marital status, household size, contact with others, education, labour force status, current smoking, alcohol consumption, chronic conditions, ADL and instrumental activities of daily living (IADL), excluding the variable itself; The higher the scores for the three health outcomes indicate more severe depressive symptoms, greater life satisfaction, and better self-reported health status. The colour of each cell indicates the effect size between extreme wealth loss and mental health outcomes. The single asterisk indicates that the association of extreme wealth loss with mental well-being is statistically significant.

**Explanation of authorship change**

During production review, several differences were identified between the authorship byline in the initially submitted manuscript and that in the latest revised version. We provide the following clarification.

First, Ke Peng and Minzhi Xu were indicated as joint first authors in the initially submitted manuscript. This contribution note was inadvertently omitted during preparation of the latest revised version and has now been restored/clarified to maintain consistency with the original authorship contribution.

Second, Yun Liu was added as an author at the revision stage. Yun Liu was not involved in the initial preparation or submission of the manuscript. However, after peer review, the authors identified the need for additional methodological refinement, further interpretation of the revised analyses, and supplementary data to adequately address the reviewers' comments. Yun Liu provided important intellectual input on the revision strategy, contributed newly collected data that were incorporated into the revised manuscript, helped refine the presentation and interpretation of the revised analyses, and critically revised the manuscript for important intellectual content. The authors therefore agreed that Yun Liu met the criteria for authorship.

Third, the corresponding authorship was updated to include Yun Liu and Yanhong Gong as co-corresponding authors. The revised manuscript also identifies Yun Liu and Yanhong Gong as joint senior authors, reflecting their respective contributions to supervision, data-related support, critical revision, and final oversight of the revised manuscript.

All authors have reviewed and approved the revised authorship byline, authorship notes, and authorship contribution statement. All authors agree that these changes accurately reflect the actual scientific contributions made to the manuscript.
